# Should central venous catheter be systematically removed in patients with suspected catheter related infection?

**DOI:** 10.1186/s13054-014-0564-3

**Published:** 2014-10-17

**Authors:** Leonardo Lorente, María M Martín, Pablo Vidal, Sergio Rebollo, María I Ostabal, Jordi Solé-Violán, Working Group on Catheter Related Infection Suspicion Management of GTEIS/SEMICYUC

**Affiliations:** Intensive Care Unit, Hospital Universitario de Canarias, Ofra s/n, La Laguna, Santa Cruz de Tenerife, 38320 Spain; Intensive Care Unit, Hospital Universitario Nuestra Señora Candelaria, Carretera Rosario s/n, Santa Cruz Tenerife, 38010 Spain; Intensive Care Unit, Complexo Hospitalario Universitario de Ourense, C/ Ramon Puga Noguerol n°54, Ourense, 32005 Spain; Intensive Care Unit, Hospital General Universitario Santa Lucía, C/ Mezquita s/n, Paraje Los Arcos, Cartagena, Murcia 30202 Spain; Intensive Care Unit, Hospital Miguel Servet, Paseo Isabel la Catolica n° 1-3, Zaragoza, 50009 Spain; Intensive Care Unit, Hospital Universitario Dr. Negrín, CIBERES, Barranco de la Ballena s/n, Las Palmas de Gran Canaria, 35010 Spain

## Abstract

**Introduction:**

Best clinical practice for patients with suspected catheter-related infection (CRI) remains unclear according to the latest Infectious Diseases Society of America (IDSA) guidelines. Thus, the objective of this study was to analyze clinical practice concerning the central venous catheter (CVC) and its impact on prognosis in patients with suspected CRI.

**Methods:**

We performed a prospective, multicenter, observational study in 18 Spanish Intensive Care Units (ICUs). Inclusion criteria were patients with CVC and suspected CRI. The following exclusion criteria were used: age less than 18 years; pregnancy; lactation; human immunodeficiency virus; neutropenia; solid or haematological tumor; immunosuppressive or radiation therapy; transplanted organ; intravascular foreign body; haemodynamic instability; suppuration or frank erythema/induration at the insertion site of the CVC, and patients with bacteremia or fungemia. The end-point of the study was mortality at 30 days of CRI suspicion.

**Results:**

The study included 384 patients. In 214 (55.8%) patients, CVC was removed at the moment of CRI suspicion, in 114 (29.7%) CVC was removed later and in 56 (14.6%) CVC was not removed. We did not find significant differences between survivors (n =311) and non-survivors (n =73) at 30 days according to CVC decision (*P* =0.26). The rate of confirmed catheter-related bloodstream infection (CRBSI) was higher in survivors than in non-survivors (14.5% versus 4.1%; *P* =0.02). Mortality rate was lower in patients with CRBSI than in the group of patients whose clinical symptoms were due to other causes (3/48 (6.25%) versus 70/336 (20.8%); *P* =0.02). We did not find significant differences in mortality in patients with confirmed CRBSI according to CVC removal at the moment of CRI suspicion (n =38) or later (n =10) (7.9% versus 0; *P* =0.99).

**Conclusion:**

In patients with suspected CRI, immediate CVC removal may be not necessary in all patients. Other aspects should be taken into account in the decision-making, such as vascular accessibility, the risk of mechanical complications during new cannulation that may be life-threatening, and the possibility that the CVC may not be the origin of the suspected CRI.

## Introduction

Clinical practice guidelines for the management of intravascular catheter-related infection (CRI) by the Infectious Diseases Society of America (IDSA) are unclear on what strategy to adopt in patients with central venous catheter (CVC) and suspected CRI [1]. There are arguments in favour of and against immediate CVC removal on suspicion of CRI. On the one hand, catheter-related bloodstream infection (CRBSI) has been associated with increased mortality [2] and delayed CVC removal could lead to worse prognosis if the focus of infection is the CVC itself [3]. On the other hand, there are arguments against immediate CVC removal when CRI is suspected.

First, one reason to suspect CRI is the presence of fever, but critically ill patients frequently develop fever and the cause is not always CRBSI; there are many other causes of fever, including non-infectious processes (such as pancreatitis, pulmonary infarction or acute respiratory distress syndrome, et cetera) and infectious processes (pneumonia, urinary tract infection and central nervous system infection) [4]. Second, the incidence of CRBSI has decreased due to the implementation of evidence-based clinical practice during CVC insertion and maintenance [5,6]. Third, in a randomized clinical trial involving 64 patients with suspected CRI, there were no differences in outcome between groups with early CVC removal and those with watchful waiting; however, in the watchful-waiting group, only 38% underwent catheter removal [7]. Fourth, vascular catheterization by new puncture entails the risk of serious and even life-threatening mechanical complications such as vascular lesion, haematoma, haemothorax, pneumothorax, nerve injury and gas embolism [8]. The objective of this study was to analyze clinical practice for CVC management in critically ill patients with suspected CRI, and its impact on patient prognosis.

## Methods

### Design and subjects

We performed a prospective, observational, multicentre study in 18 Spanish ICUs. The study was approved by the Institutional Ethic Review Boards of the 18 participating hospitals (Review Boards are listed in Acknowledgements). Written informed consent from the patients or from their family members was obtained.

Inclusion criteria were ICU patients with CVC and suspected CRI. CRI was suspected when a patient developed a new episode of fever or sepsis. Fever was considered as temperature ≥38°C. Sepsis was defined according to the International Sepsis Definitions Conference criteria [9]. Exclusion criteria were: age <18 years, pregnancy, lactation, HIV, neutropenia (<1000/mm3), solid or haematological tumor, immunosuppressive or radiation therapy, transplanted organ, intravascular foreign body (for example, pace-maker, prosthetic heart valve), haemodynamic instability (start of norepinephrine to maintain adequate blood pressure or increase of dose with 0.25 μg · kg · min over the preceding 12 h), suppuration or frank erythema/induration at the insertion site of the CVC, patients with bacteraemia or fungemia, and tunneled catheters. The decision about choice of immediate CVC removal or watchful waiting was made by the physician responsible for each patient.

### Variables recorded

The following variables were recorded for each patient: Age, sex, diagnosis on admission, diabetes mellitus, chronic obstructive pulmonary disease (COPD), steroids, antibiotics, tracheostomy, temperature, leukocytes, neutrophils, pressure of arterial oxygen/fraction inspired of oxygen (PaO_2_/FIO_2_), platelets, activated partial thromboplastin time (aPTT), international normalized ratio (INR), sepsis-related organ failure assessment (SOFA) score [10], lactic acid, bilirubin, creatinine, CVC decision at moment of CRI suspicion, moment of CVC removal, and cause of symptoms.

### End point

The end point of the study was mortality at 30 days after suspicion of CRI.

### Definitions

CVC-related bacteraemia (CVCB) was defined as a positive blood culture by recognized pathogen (or two positive blood cultures by skin contaminant microorganism) obtained from a peripheral vein, and catheter-tip colonization or positive conservative cultures for diagnosis of CVCB (superficial cultures, quantitative blood cultures or differential time to positivity) with the same organism as the blood culture (the same species and identical antimicrobial susceptibility).

Catheter-tip colonization was considered as significant growth of a microorganism on the CVC tip (>15 colony-forming units). Superficial cultures were considered to be positive when the same microorganism (>15 colony-forming units per plate) was isolated in cultures of skin and/or catheter hubs and in peripheral blood. Quantitative blood cultures were defined as positive when the number of colony-forming units of microorganisms isolated per milliliter of catheter-drawn blood was at least three times greater than that of blood obtained from a peripheral vein. Differential time to positivity was defined as positive when the blood through any of the CVC hubs yielded positive results at least 120 minutes earlier than the positivity of a blood sample drawn simultaneously from a peripheral vein. Primary bacteraemia (PB) was defined as a positive blood culture obtained from a peripheral vein, no apparent source of bacteraemia and disappearance of symptoms within 48 hours after removal of the venous catheter. CRBSI included the presence of CVCB or PB.

### Statistical analysis

Continuous variables are reported as means and standard deviations, and categorical variables as frequencies and percentages. We used the Mann-Whitney *t*-test to compare continuous variables between groups. Comparison of categorical variables between groups was performed using the chi-square test. We carried out a propensity analysis with logistic regression to control for the effect of sex, admission diagnosis, COPD, SOFA score and CRBSI as the cause of clinical symptoms at the moment of CVC removal. The dependent variable was moment of CVC removal, and the independent variables were sex, admission diagnosis, COPD, SOFA score and CRBSI as the cause of the symptoms. To control for the confounding impact of propensity scores in mortality, we included propensity scores jointly with moment of CVC removal in a binomial regression model. Risk ratio and 95% confidence intervals were calculated as measures of the clinical impact of the predictor variables. *P*-values <0.05 were considered statistically significant. Statistical analysis was performed with SPSS 17.0 (SPSS Inc., Chicago, IL, USA).

## Results

The study included 384 patients with CVC and suspected CRI, of whom 73 (19.0%) had died at 30 days of suspected CRI. The causes of clinical symptoms were as follows: 48 patients (12.5%) with CRBSI, 101 (26.0%) with pneumonia, 60 (15.6%) with tracheobronchitis, 27 (7.0%) with urinary infection, 21 (5.5%) with skin infection, 2 (0.5%) with osteomyelitis, 4 (1.0%) with central nervous system infection, 27 (7.0%) with abdominal infection, 1 (0.3%) with otitis, 7 (1.8%) with non-infectious causes, 86 (22.4%) with unknown causes. The seven patients with non-infectious causes had the following: pancreatitis (n =3), mesenteric ischemia (n =2), deep venous thrombosis (n =1) and pulmonary thromboembolism (n =1).

**Figure 1:**
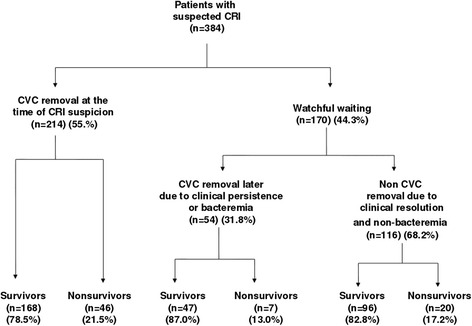
**Characteristics of patients with suspected catheter-related infection (CRI) according to decision on removal of central venous catheter (CVC) and 30-day mortality.**

Figure 1 shows the flowchart of patients included in the study regarding CVC decision and 30-day mortality. In 214 patients (55.8%) the CVC was removed immediately on suspicion of CRI. In 170 (44.3%) patients the strategy of watchful waiting was adopted, with CVC maintained and microbiology cultures performed. Of the 214 patients whose CVC was removed immediately on suspicion of CRI, in 180 patients a new CVC was canalized and in 34 patients a new CVC catheter was not needed; and of those 214 patients, 46 (21.5%) had died at 30 days after suspected CRI. Of the 170 patients whose CVC was maintained along with microbiology cultures, in 56 patients conservative cultures were performed for the diagnosis of CRBSI (superficial cultures, quantitative blood cultures or differential time to positivity) and in 114 patients these conservative cultures were not performed. And in those 170 patients whose CVC was initially maintained, the CVC was removed later in 54 patients, 7 (13.0%) had died at 30 days. In 116 patients the CVC was not removed and 20 (17.2%) had died at 30 days after suspected CRI.

In the 54 patients in whom the catheter was not initially removed, the reasons for later CVC removal were the following: 45 (83.3%) had persistent fever, 5 (9.3%) persistent sepsis, 3 (5.6%) bloodstream infections and 1 (1.9%) had suppuration at the insertion site. Mean time from suspected CRI to catheter removal was 3.9 ± 2.5 days.

Table 1 shows comparisons of demographic and clinical parameters between 30-day surviving (n =311) and non-surviving (n =73) patients at the moment of suspected CRI. We found that 30-day non-surviving patients showed higher SOFA score and lactic acid levels than survivors at the time of suspected CRI. We did not find significant differences between survivors and non-survivors in CVC decision (*P* =0.26) and the moment of CVC removal (*P* =0.31). In addition, the rate of confirmed CRBSI was higher in 30-day survivors than in non-survivors (14.5% versus 4.1%; *P* =0.02).

**Table 1:** **Characteristics of 30-day survivor and non-survivor patients**

**Data**	**Survivors (n =311)**	**Non-survivors (n =73)**	***P*** **-value**
Age, years, median (percentile 25 to 75)	61 (50 to 71)	71 (59 to 76)	0.44
Sex, female, n (%)	210 (67.5)	43 (58.9)	0.17
Admission diagnostic, n (%)			0.15
Medical	203 (65.3)	56 (76.7)	
Surgical	81 (26.0)	14 (19.2)	
Traumatology	27 (8.7)	3 (4.1)	
Diabetes mellitus, n (%)	75 (24.1)	22 (30.1)	0.30
Chronic obstructive pulmonary disease, n (%)	39 (12.5)	17 (23.3)	0.03
Corticosteroids, n (%)	55 (17.7)	17 (23.3)	0.32
Antibiotics, n (%)	225 (72.3)	56 (76.7)	0.56
Tracheostomy, n (%)	54 (17.4)	17 (23.3)	0.24
Temperature, °C, median (percentile 25 to 75)	38.0 (37.8 to 38.4)	38.4 (38.0 to 38.5)	0.44
Leukocytes, median*10^3^/mm^3^ (percentile 25 to 75)	10.6 (8.2 to 15.4)	12.6 (8.5 to 19.5)	0.01
Neutrophils, median*10^3^/mm^3^ (percentile 25 to 75)	8.2 (5.9 to 12.6)	10.0 (6.8 to 14.8)	0.18
Pa0_2_/FI0_2_ ratio, median (percentile 25 to 75)	225 (177 to 294)	190 (110 to 331)	0.26
Platelets, median*10^3^/mm^3^ (percentile 25 to 75)	225 (116 to 317)	187 (63 to 217)	0.16
aPTT, seconds, median (percentile 25 to 75)	29 (26 to 30)	31 (27 to 34)	0.61
International normalized ratio, median (percentile 25 to 75)	1.1 (1.0 to 1.2)	1.1 (1.0 to 1.1)	0.86
SOFA score, median (percentile 25 to 75)	5 (4 to 8)	8 (5 to 13)	<0.001
Lactic acid, mmol/L, median (percentile 25 to 75)	1.2 (0.9 to 1.6)	1.7 (1.0 to 2.3)	<0.001
Bilirubin, mg/dl, median (percentile 25 to 75)	0.6 (0.4 to 0.9)	0.7 (0.3 to 2.3)	0.40
Creatinine, mg/dl, median (percentile 25 to 75)	0.8 (0.6 to 1.4)	0.7 (0.5 to 1.0)	0.57
CVC decision at moment of CRI suspicion, n (%)			0.26
CVC removal without new catheter	28 (9.0)	6 (8.2)	
CVC removal with new catheter	140 (45.0)	40 (54.8)	
Watchful waiting without conservative cultures for diagnosis of CRBSI	99 (31.8)	15 (20.5)	
Watchful waiting with conservative cultures for diagnosis of CRBSI	44 (14.1)	12 (16.4)	
Moment of CVC removal, n (%)			0.31
No CVC removal	96 (30.9)	20 (27.4)	
CVC removal at moment of CRI suspicion	168 (54.0)	46 (63.0)	
CVC removal later at moment of CRI suspicion	47 (15.1)	7 (9.6)	
Cause of symptomatology, n (%)			0.03
CRBSI	45 (14.5)	3 (4.1)	
Pneumonia	69 (22.2)	32 (43.8)	
Tracheobronchitis	53 (17.0)	7 (9.6)	
Urinary infection	22 (7.1)	5 (6.8)	
Skin infection	15 (4.8)	6 (8.2)	
Osteomyelitis	2 (0.6)	0	
Central nervous system infection	3 (1.0)	1 (1.4)	
Abdominal infection	22 (7.1)	5 (6.8)	
Otitis	1 (0.3)	0	
Non-infectious	5 (1.6)	2 (2.7)	
Unknown	74 (23.8)	12 (16.4)	
CRBSI as cause of symptomatology, n (%)	45 (14.5)	3 (4.1)	0.02

PaO_2_/FIO_2_, pressure of arterial oxygen/fraction inspired of oxygen; aPTT, activated partial thromboplastin time; SOFA, sepsis-related organ failure assessment; CVC, central venous catheter; CRI, catheter-related infection; CRBSI, catheter-related bloodstream infection.

Mortality rate at 30 days after suspected CRI was lower in patients with CRBSI than in the group of patients whose clinical symptoms were due to other causes (3/48 (6.25%) versus 70/336 (20.8%); *P* =0.02). However, there were no statistically significant differences in SOFA score (5 (2 to 7) versus 5 (3 to 8); *P* = 0.48) or lactic acid levels (1.3 (1.0 to 1.9) versus 1.2 (0.9 to 1.8); *P* =0.74) at the time of suspected CRI when comparing patients with CRBSI and the group of patients whose clinical symptoms were due to other causes. Table 2 shows higher risk of mortality in patients with immediate CVC removal on suspicion of CRI, compared with later removal of CVC (OR 3.97, 95% CI 1.30, 12.10; *P* =0.02).

**Table 2:** **Logistic regression model to predict survival at 30 days**

	**Odds ratio**	**95% Confidence interval**	***P*** **-value**
Moment of CVC removal	-	-	0.04
CVC removal immediately on CRI suspicion versus	2.80	0.87-8.98	0.08
No CVC removal			
CVC removal immediately on CRI suspicion versus	3.97	1.30, 12.10	0.02
CVC removal later			
No CVC removal versus	1.42	0.76, 2.65	0.27
CVC removal later			
Propensity score 1	0.001	(0.0001, 0.39)	0.02
(Reference category: no CVC removal)			
CVC removal immediately on CRI suspicion			
Propensity score 2	0.001	(0.0001, 0.07)	0.001
(Reference category: no cvc removal)			
CVC removal later at moment of CRI suspicion			

CVC, central venous catheter; CRI, catheter-related infection.

ICU may be a confounder for the association between moment of CVC removal and mortality. However, in the chi squared test between ICU and mortality, χ^2^ was 27.1 (degrees of freedom 17; *P* =0.057). Thus, ICU cannot be considered a confounder for the association between moment of CVC removal and mortality.

Table 3 shows comparisons of demographic and clinical parameters of patients with CRBSI confirmed according to immediate CVC removal on suspicion of CRI (n =38) or later (n =10). The group with immediate CVC removal had higher aPTT and rates of diabetes mellitus. We did not find statistically significant differences in mortality between the two groups (7.9% versus 0; *P* =0.99).

**Table 3:** **Characteristics of patients with CRBSI according to moment of CVC removal**

**Data**	**CVC removed at moment of suspicion of CRI (n =38)**	**CVC removed later after suspicion of CRI (n =10)**	***P*** **-value**
Age, years, median (percentile 25 to 75)	63 (53 to 72)	57 (49 to 64)	0.30
Sex, female, n (%)	11 (28.9)	5 (50.0)	0.27
Admission diagnostic, n (%)			0.10
Medical	20 (52.6)	8 (80.0)	
Surgical	17 (44.7)	1 (10.0)	
Traumatology	1 (2.6)	1 (10.0)	
Diabetes mellitus, n (%)	8 (21.6)	6 (60.0)	0.04
COPD, n (%)	5 (13.2)	2 (20.0)	0.63
Corticosteroids, n (%)	10 (26.3)	3 (30.0)	0.99
Antibiotics, n (%)	28 (73.7)	6 (60.0)	0.45
Tracheostomy, n (%)	12 (31.6)	2 (20.0)	0.70
Temperature, °C, median (percentile 25 to 75)	38.2 (37.9 to 38.8)	38.1 (37.5 to 38.5)	0.32
Leukocytes, median*10^3^/mm^3^ (percentile 25 to 75)	12.5 (8.9 to 16.5)	14.6 (6.9 to 20.1)	0.68
Neutrophils, median*10^3^/mm^3^ (percentile 25 to 75)	8.6 (6.1 to 13.1)	11.0 (4.6 to 14.4)	0.77
Pa0_2_/FI0_2_ ratio, median (percentile 25 to 75)	271 (200 to 320)	260 (190 to 347)	0.96
Platelets, median*10^3^/mm^3^ (percentile 25 to 75)	192 (130 to 299)	191 (110 to 378)	0.99
aPTT, seconds, median (percentile 25 to 75)	32 (28 to 38)	26 (25 to 27)	0.03
INR, median (percentile 25 to 75)	1.09 (1.01 to 1.20)	1.20 (1.10 to 1.24)	0.11
SOFA score, median (percentile 25 to 75)	6 (2 to 8)	4 (3 to 6)	0.61
Lactic acid, mmol/L, median (percentile 25 to 75)	1.28 (0.95 to 1.60)	1.40 (0.88 to 2.47)	0.65
Bilirubin, mg/dl, median (percentile 25 to 75)	1.0 (0.6 to 2.9)	0.6 (0.4 to 1.3)	0.25
Creatinine, mg/dl, median (percentile 25 to 75)	0.9 (0.6 to 1.3)	1.3 (0.6 to 1.9)	0.39
Mortality, n (%)	3 (7.9)	0	0.99

COPD, chronic obstructive pulmonary disease; PaO_2_/FIO_2_, pressure of arterial oxygen/fraction inspired of oxygen; aPTT, activated partial thromboplastin time; INR, international normalized ratio; SOFA, sepsis-related organ failure assessment; CVC, central venous catheter; CRBSI, catheter-related bloodstream infection.

New CVCs were placed in 303 patients as follows: 162 subclavian, 61 femoral, and 59 jugular catheters, and 21 peripherally inserted central catheters (PICC). We recorded two cases of pneumothorax in 162 patients with subclavian access (1.2%), without death.

Regarding the cause of CRBSI, we found 32 Gram-positive coccus, 15 Gram-negative bacilli and one yeast. The microorganisms responsible for CRBSI were as follows: four *Staphylococus aureus*, nineteen coagulase-negative *Staphylococcus spp.*, one *Streptococcus pyogenes*, one *Streptococcus viridans*, five *Enterococcus faecalis*, two Enterococcus faecium, one *Escherichia coli*, two *Klebsiella spp*., one *Enterobacter spp*., one *Serratia marcescens*, seven *Pseudomonas aeruginosa*, three *Acinetobacter spp*., and one *Candida glabrata*. Two patients with CRBSI due to coagulase-negative *Staphylococus spp*., and one due to *E. faecalis* had died at 30 days of suspected CRI.

## Discussion

The most interesting findings of our study were that CRBSI was confirmed in only 12% of patients with suspected CRI, that mortality due to CRBSI was lower than that due to other causes explaining the clinical symptoms, that the group of patients with immediate CVC removal on suspicion of CRI showed a higher rate of mortality at 30 days of suspected CRI than the group with later CVC removal, and in patients with confirmed CRBSI there was no difference in mortality between immediate or later CVC removal on suspicion of CRI.

In our study, an infection was the cause of the clinical symptoms in 76% of cases. This percentage is higher than those in previous series reporting that infectious events were the cause of fever in critically ill patients, ranging between 17% [11] and 50% [12-14] of cases. We found that CRBSI was confirmed in 12% of cases with suspected CRI. This rate in our study could be due to the implementation of the Spanish Bacteremia Zero project [6]. After the previous success of the program pioneered by the Johns Hopkins Quality and Safety Research Group in Michigan (Keystone ICU project) which was associated with a dramatic and sustained reduction of CRBSI [5], the Bacteremia Zero project aimed to assess its effectiveness after contextual adaptation and large-scale implementation in Spanish ICUs. In 2008, a collaborative agreement of the Spanish Ministry of Health, Social Policy and Equality, the Patient Safety Programme of the World Health Organization (WHO), the Spanish Society of Intensive and Critical Care Medicine and Coronary Units (SEMICYUC), and the Johns Hopkins Quality and Safety Research Group (now the Armstrong Institute for Patient Safety and Quality) was established to implement the Keystone project in Spanish ICUs. CRBSI was reduced from 3.07 to 1.12 episodes per 1,000 catheter-days.

One interesting finding of our study was that mortality due to CRBSI was lower than that in the group of patients whose clinical symptoms were explained by another cause (6.25% versus 20.8%). Another interesting finding was that there were no differences between survivors and non-survivors in terms of CVC decision and moment of CVC removal, nor in mortality between immediate CVC removal on suspicion of CRI or later in patients with confirmed CRBSI. These findings are in consonance with the results of the study by Rijnders *et al*. [7]. In that randomized clinical trial involving 64 patients with suspected CRI, the authors compared the outcomes of patients with early removal of short-term CVC or watchful waiting. There were no differences in mortality, duration of ICU stay or resolution of fever between the two groups of patients. However, in the early removal group all catheters were removed and in the watchful waiting group only 38% catheters were removed.

We found a low rate of mechanical complications during new puncture for vascular catheterization. Only 1.2% presented with pneumothorax due to catheterization of the subclavian vein, and this rate is within the 0 to 6% range previously published [8].

Taking into account all the above findings, we believe that in patients with haemodynamic stability, and without history of immunosuppressive disease or therapy, intravascular foreign body or transplanted organ, and without suppuration/inflammation at the insertion site or bacteraemia/fungemia, the immediate CVC removal may be unnecessary when CRI is suspected. And if none of these conditions are present, the strategy of watchful waiting for microbiological results before CVC removal could be adopted. This approach is in consonance with the opinion of other authors [15].

In addition, we believe the decision on immediate CVC removal or watchful waiting in patients with suspected CRI should take into account the following aspects for each patient: first, vascular accessibility, as new vascular catheterization may be very difficult in some cases due to poor vascular access; second, the risk of mechanical complications during new canalization that may even be life-threatening (for example, patients with coagulopathy or severe respiratory disease); third, the possibility that the CVC may not be the origin of the suspected CRI. In this regard, CVCs placed in the jugular vein of tracheostomized patients [16] or those placed in the femoral vein present a higher risk of CRBSI [17]; however, the risk of CRBSI decreases with the use of antimicrobial-impregnated catheters [18] and dressings [19].

The strengths of our study are that it was a multicentre study (which increases the applicability of its results to other ICUs) and the larger sample size compared with the study by Rijnders *et al*. (384 versus 64 episodes of suspected CRI) [7]. However, it has certain limitations. First, the practice of immediate CVC removal or watchful waiting was not subject to random assignment. The decision on which strategy to adopt was made by the patient’s physician in all cases; however, we tried to control for this limitation with a propensity analysis. Second, some patients were excluded and it could be interesting to analyze optimal clinical practice in these patients. Third, we have not reported the proportion of ICU patients with central venous catheters who were excluded.

## Conclusion

In patients with suspected CRI, immediate CVC removal may be unnecessary if the patient does not have a transplanted organ, intravascular foreign body, haemodynamic instability, immunosuppressive disease or therapy, suppuration or inflammation at the insertion site, or bacteraemia or fungemia. Other aspects should be taken into account in the decision-making, such as vascular accessibility, the risk of mechanical complications during new cannulation that may be life-threatening, and the possibility that the CVC may not be the origin of the suspected CRI.

## Key messages

- Mortality due to CRBSI was lower than that due to other causes explaining the clinical symptoms
- We did not find significant differences in mortality in patients with confirmed CRBSI according to immediate CVC removal on suspicion of CRI or later
- In patients with suspected CRI, immediate CVC removal may be unnecessary
- Other aspects should be taken into account in the decision-making

## Abbreviations

aPTT: activated partial thromboplastin time
COPD: chronic obstructive pulmonary disease
CRBSI: catheter-related bloodstream infection
CRI: catheter-related infection
CVC: central venous catheter
IDSA: Infectious Diseases Society of America
INR: international normalized ratio
PaO_2_/FIO_2_: pressure of arterial oxygen/fraction inspired of oxygen
SOFA: sepsis-related organ failure assessment
